# Evaluation of the Efficacy of Caudal Epidural Neuroplasty in Patients With Lumbar Epidural Fibrosis

**DOI:** 10.7759/cureus.52606

**Published:** 2024-01-20

**Authors:** Ferhat Ege

**Affiliations:** 1 Pain Management, Gazi Yasargil Training and Research Hospital, Diyarbakır, TUR

**Keywords:** epidural lysis, chronic pain management, scar tissue, neuroplasty, epidural fibrosis

## Abstract

Introduction

Lumbar and leg pain can be caused by many factors, including scar tissue in the epidural space. Epidural fibrosis may cause chronic radicular low back pain. Adhesions in the epidural space may occur due to surgical or non-surgical reasons. Epidural adhesiolysis, i.e., neuroplasty, eliminates the pain-causing effects of scar tissue by releasing the nerve from the scar tissue or decompressing the nerve. In light of this information, this study was conducted to evaluate the effectiveness of percutaneous epidural neuroplasty interventions performed in the algology clinic in patients with lumbar epidural fibrosis who have and have not undergone lumbar surgery.

Methods

The sample of this retrospective study consisted of 72 patients with chronic radicular low back pain, finding fibrosis in the epidural region on contrast-enhanced magnetic resonance imaging (MRI), filling defect after epidurogram, and caudal epidural neuroplasty. Patients' visual analog scale (VAS) and Oswestry disability index (ODI) scores, pregabalin, duloxetine, and opioid doses were evaluated before and one month and six months after having neuroplasty.

Results

The VAS and ODI scores and pregabalin, duloxetine, and opioid doses decreased significantly in patients who had had caudal epidural neuroplasty at post-procedure endpoints compared to before the procedure (p<0.001). The paired temporal comparisons of the data of the patients who underwent epidural neuroplasty procedures before the procedure, one month after the procedure, and six months after the procedure revealed significant differences in the VAS and ODI scores (p<0.001). Additionally, the analysis of patients' VAS scores revealed that the pre-procedure VAS scores decreased significantly more one month after the procedure in patients without a history of lumbar surgery than in patients with a history of lumbar surgery.

Conclusions

The findings of our study demonstrated that fluoroscopy-guided percutaneous epidural neuroplasty alleviated pain and improved physical functions and quality of life. In conclusion, percutaneous epidural neuroplasty is a safe and effective treatment method for patients with lumbar epidural fibrosis.

## Introduction

Epidural fibrosis may cause chronic radicular lower back pain, negatively affecting patients' quality of life. Adhesions in the epidural space may occur due to surgical or non-surgical causes. Non-surgical causes include annular tear, infection, hematoma, and administration of intrathecal contrast material [1]. Scar tissue may develop in the ventral, dorsal, and lateral regions of the epidural space. Dorsal epidural fibrosis may develop due to surgical hematoma absorption, ventral epidural fibrosis may develop due to disc defects, and lateral epidural fibrosis may develop due to disc defects, facet hypertrophy, and lateral foraminal stenosis [2]. In the neural foramen, epidural veins accompany the nerve roots. Epidural scar tissue causes compression of the veins, which gives rise to edema in the epidural area [3]. Stand-alone epidural fibrosis is not a cause of pain. Epidural fibrosis-induced scar tissue fixes the nerve root in one position, causing inflammation of the nerve root. Inflammation causes stretching and compression of the nerve root and increased pain during movement [4]. Diagnosis of epidural fibrosis is made by physical examination and radiographic methods, including magnetic resonance imaging (MRI), computed tomography (CT), epidurography, and epidural endoscopy. The gold standard diagnostic method is epidural endoscopy [1]. Epidural neuroplasty, also known as epidural lysis, can be used to treat epidural fibrosis. Epidural lysis is commonly performed in patients with radiculopathy and nerve root compression caused by epidural scar tissue. The objective of lysis of adhesion areas in neuroplasty is to deliver the drug to the target areas by opening up the scar tissue in order to suppress inflammation [2]. An epidural adhesiolysis exercise protocol (neural flossing) is recommended for patients after the procedure. The aim of the exercise protocol is to activate the nerve roots after the epidural neuroplasty procedure by "sliding" them in and out of the foramen [2].

In this context, this study was conducted to comparatively evaluate the effectiveness of percutaneous epidural neuroplasty interventions performed in the algology clinic in patients with lumbar epidural fibrosis who have and have not undergone lumbar surgery.

## Materials and methods

Research design 

This study was designed as a retrospective cohort study. The study protocol was approved by the Ethics Committee of the University of Health Sciences (01/2023-03). The study was conducted in accordance with the principles set forth in the Declaration of Helsinki.

Population and sample

The sample of this retrospective study consisted of 76 patients with chronic radicular low back pain, finding fibrosis in the epidural region on contrast-enhanced MRI, filling defect after epidurogram, and had caudal epidural neuroplasty at Hatay Training and Research Hospital Algology Clinic between October 2020 and January 2023. Four patients were excluded because the procedure could not be completed due to complications. In our study, 72 patients were analyzed. Epidural adhesiolysis was not planned for sepsis, chronic infection, patients with a history of lumbar surgery up to three months before the procedure, coagulopathy, active local infection at the procedure site, presence of syrinx in the spinal cord, presence of arachnoiditis and for patients who refused the procedure. The evaluation of patients with chronic waist and leg pain, planning of their treatments, and their percutaneous epidural neuroplasty interventions were carried out by the same experienced specialist algologist in the algology clinic.

Data collection

Patients’ pain levels and functionality were assessed at three endpoints, i.e., before and one month and six months after having neuroplasty using the visual analog scale (VAS) and Oswestry disability index (ODI), respectively. Additionally, the opioid, duloxetine, and pregabalin doses used by the patients at the study’s endpoints were obtained from the hospital records and evaluated comparatively.

Functionality

Patients’ functionality was assessed using ODI, which consists of 10 items that measure the severity of pain, personal care, lifting, walking, sitting, standing, social life, sleeping, travel status, and cognitive status of pain. Each item is assigned a score between 0 points and 5 points. The total ODI score is calculated by multiplying the scores obtained from the items by two and is expressed as a percentage value. The higher the total ODI score, the lower the functional competence and the higher the disability [5].

Pain

Patients’ pain levels were assessed using the VAS. In the VAS, patients rate their pain on a scale between 0 and 10 [6]. The higher the VAS score, the greater the severity of pain.

Radiological Evaluation

Lumbar surgery history, a finding of epidural fibrosis, the presence of recurrent or newly developing protruded or extruded herniated nucleus pulposus (HNP), and lumbar narrow canal parameters were meticulously examined based on contrast-enhanced lumbar MRI scans. A device with a magnetic field of 1.5 Tesla and a gradient power of 32 m Tesla was used for magnetic resonance imaging (Intera, Philips Healthcare, Best, Netherlands).

Procedure

**Figure 1:**
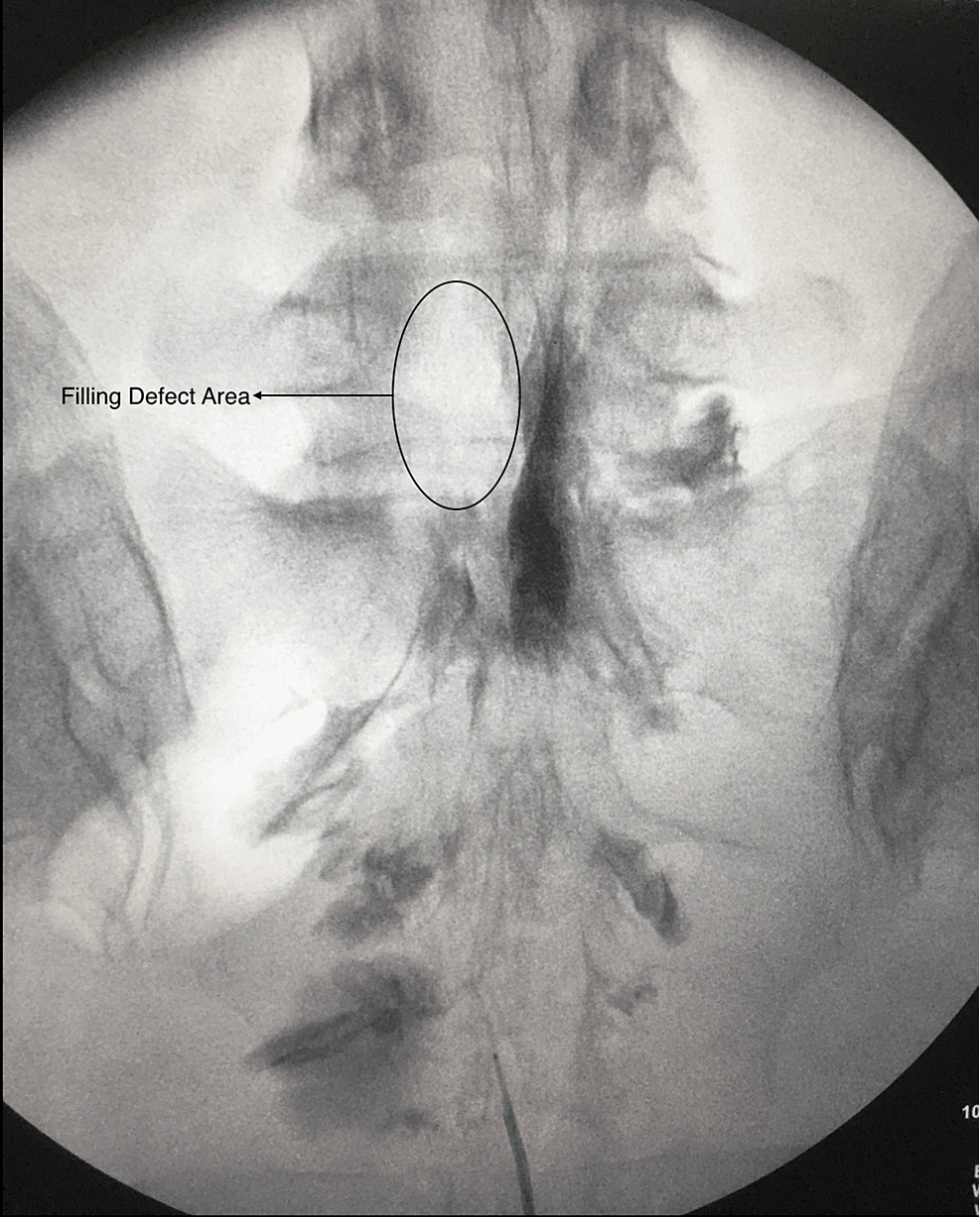
Kaudal epidurogram Filling defect at the left L5 level after caudal epidurogram.

**Figure 2:**
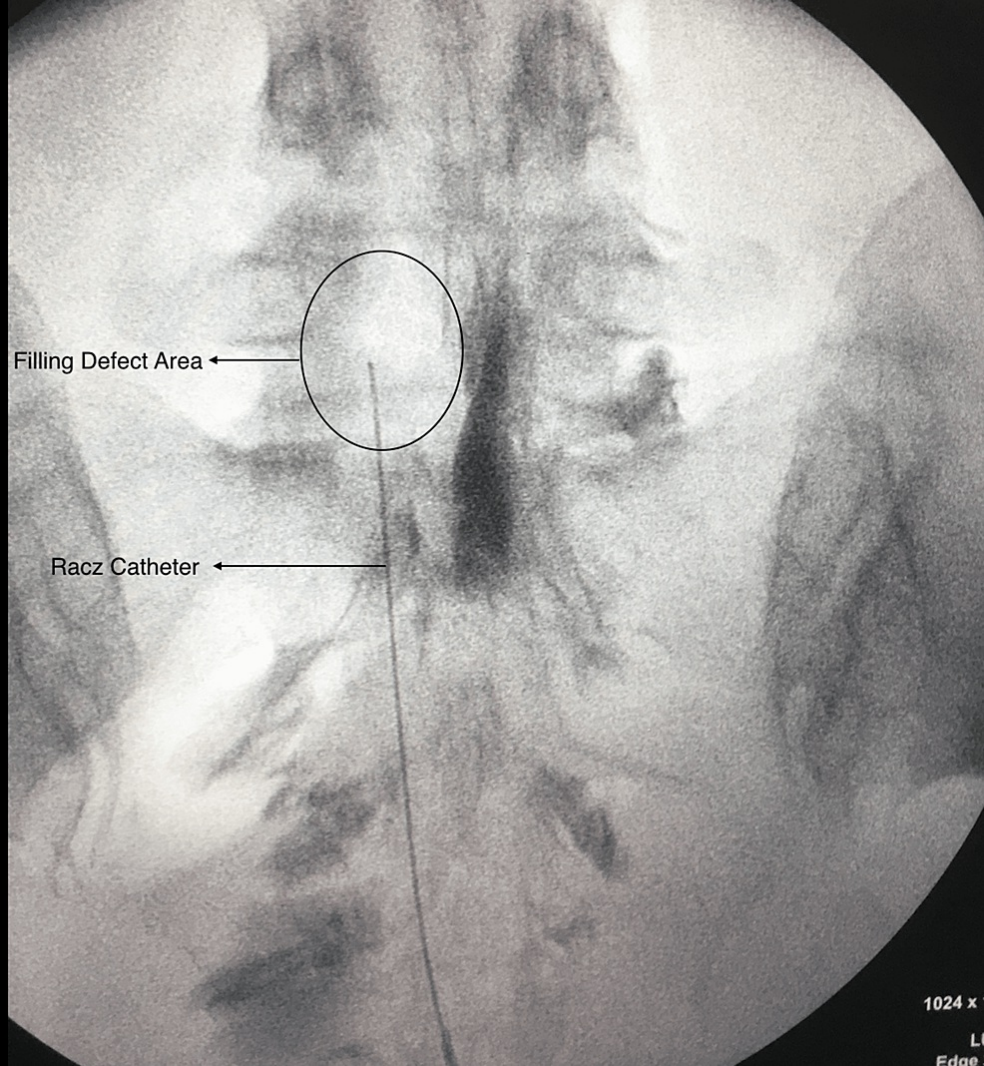
Racz catheter advancement Racz catheter is advanced toward the region of the filling defect in the anterolateral epidural area.

**Figure 3:**
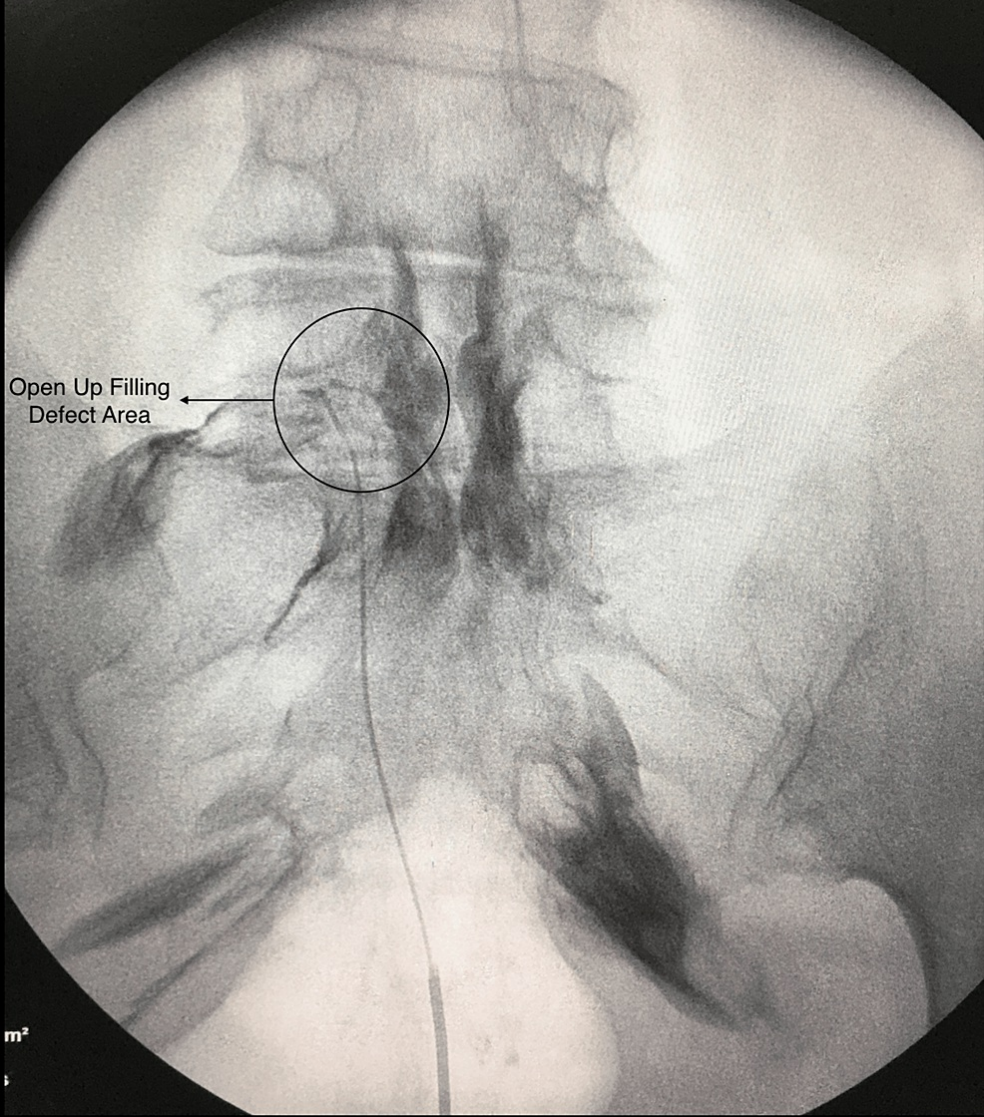
Opened up the filling defect area After giving hyaluronidase, the contrast medium is administered to open up the filling defect area.

The epidural neuroplasty was performed using the method described by Gabor et al. [2]. Patients were taken to the operating room and placed in the prone position before the procedure. Patients’ blood pressures, pulse rates, and pulse oxygen saturation levels were continuously monitored. A pillow was placed under the abdomen to correct lumbar lordosis. Patients were asked to bring their toes together and keep their heels apart. Sterilization of the procedure area was ensured. The sacral hiatus was identified under fluoroscopic guidance, and local anesthesia was achieved with 3 cc 2% lidocaine. A 16G RX Coudé (Epimed International, Dallas, TX) epidural needle was advanced into the hiatal canal under fluoroscopy. Care was taken to position the needle below the level of the S3 foramen and inside the caudal canal based on the anteroposterior (AP) fluoroscopic images to prevent possible dural rupture. Epidurogram was performed by injecting 10 mL of contrast medium, i.e., 300 mg/mL iohexol. The epidurogram showed areas of filling defect where contrast was not filled. The needle was turned so that the distal opening was ventral lateral, and then a Tun-L-Kath-guided spring catheter (Epimed International) with a bent distal tip was inserted through the needle. Guided by AP fluoroscopy, the tip of the catheter was advanced into the ventral-lateral epidural space at the level of the filling defect. The ideal position of the catheter tip was noted on AP fluoroscopic imaging in the foramen just below the mid portion of the pedicle shadow. Moreover, 2-3 mL of additional contrast medium was injected through the catheter to check for vascular, subdural, or intrathecal distribution. Once vascular distribution was determined, the catheter was repositioned. Afterward, 1,500 U hyaluronidase dissolved in 10 mL of preservative-free normal saline was injected. Additionally, 2-3 mL of contrast medium was injected, and the cicatrized tissue was opened up; 10 mL of steroid + local anesthetic + saline mixture (2 mL of 8 mg dexamethasone, 6 mL of 2% lidocaine and 2 mL saline) was slowly injected through the catheter. Then, the procedure was terminated (Figures 1-3). The patients were kept under observation for two hours after the procedure. The patients were discharged without any complications.

Statistical analysis

The statistical analyses were conducted with the Statistical Product and Service Solutions (SPSS, version 23.0; IBM Corp., Armonk, NY) software package. The descriptive statistics obtained from the research data were expressed as numbers (n) and percentage (%) values in the case of categorical variables and as mean ± standard deviation (min-max) values in the case of numerical variables. The conformity of the numerical data to the normal distribution was assessed with the Shapiro-Wilk test. The Mann-Whitney U test was used to compare the percentage differences in VAS and ODI scores over time between the patients with and without a history of lumbar surgery. The variation of the research data over time was analyzed with the Friedman test. Wilcoxon signed-rank test with Bonferroni correction was used in paired comparisons (p=0.05/3=0.016). The significance level was set at 0.05 in all other statistical tests.

## Results

The sample of the study consisted of 76 patients, 28 (%36.8) female and 48 (%63.1) male, aged between 28 and 58, who underwent epidural lysis. While 31 (%40.7) of these patients had a history of lumbar surgery, 45 (%59.2) of them did not. The procedure was terminated due to complications of intrathecal passage in three patients and catheter rupture in one patient. Descriptive characteristics of the patients are given in Table 1.

**Table 1:** Patients’ descriptive characteristics Categorical variables were expressed using frequency (n) and percentage (%) values, whereas continuous variables were expressed using mean ± standard deviation values.

Variables	n (%)/Mean±SD
Age	47.5±7.99
Gender	
Male	48 (63.1)
Female	28 (36.8)
Lumbar Surgery History	
Yes	31 (40.7)
No	45 (59.2)
Complications	
Yes	4 (5.2)
No	72 (94.7)

Statistical analysis was performed on 72 patients due to four patients who had the procedure terminated due to complications. There were statistically significant decreases in the mean VAS and ODI scores six months after the procedure compared to before the procedure (mean VAS score decreased from 8.4 to 3.6 in patients with a history of lumbar surgery and from 7.0 to 2.8 in patients without a history of lumbar surgery, and mean ODI score decreased from 88.6 to 36.5 in patients with a history of lumbar surgery and from 69 to 27.9 in patients without a history of lumbar surgery) (p<0.001) (Table 2).

**Table 2:** Patients’ pain and functionality over time Comparison of the effect of epidural lysis on VAS and ODI scores in patients with and without a surgical history. * P-values of three time-period comparisons of VAS and ODI scores. Friedman tests were used for statistical analyses. Variables were expressed using the median and min-max values. Abbreviations: VAS: visual analog scale; ODI: Oswestry disability index, Min: minimum, Max: maximum

EPIDURAL LYSIS (n: 72)					
Variables	Endpoints	LUMBAR SURGERY PATIENT WITH A HISTORY OF SURGERY (n:30)		PATIENT WITHOUT A HISTORY OF LUMBAR SURGERY (n:42)	
		Min-Max	Median	Min-Max	Median
VAS	Pre-procedure	7-9	8	6-8	7
	Post-procedure 1^st^ month	4-8	4	2-7	3
	Post-procedure 6^th^ month	3-8	3	2-8	3
P-value		p<0.001*		p<0.001*	
ODI	Pre-procedure	76-94	94	62-76	69
	Post-procedure 1^st^ month	34-90	42	28-74	33
	Post-procedure 6^th^ month	28-88	35	22-74	27
P-value		p<0.001*		p<0.001*	

No significant change was observed in the mean VAS and ODI scores of 5 (%6.94) patients. Of these five patients, who did not benefit from the procedure, four had a history of lumbar surgery, and one did not have a history of lumbar surgery. Patients who did not benefit from the procedure underwent a second epidural neuroplasty procedure one month after the first one, yet they did not benefit from the second procedure either. Additionally, the analysis of patients' VAS scores revealed that the VAS scores decreased significantly more one month after the procedure compared to before the procedure in patients without a history of lumbar surgery than in patients with a history of lumbar surgery (p<0.001). On the other hand, there was no significant difference between the patients with and without a history of lumbar surgery in the percentage difference between patients’ pre-procedure VAS scores and VAS scores six months after the procedure (0.163). There was also no significant difference between the patients with and without a history of lumbar surgery in the percentage difference between patients’ pre-procedure ODI scores and ODI scores one month after the procedure and between patients’ pre-procedure ODI scores and ODI scores six months after the procedure (p=0.473) (Table 3).

**Table 3:** Percentage comparison of changes in patients' pain levels and functionality over time Percentage comparison of changes in pain levels and functionality over time in patients with and without a surgical history. ​​​​​​​​​Variables were expressed using median and min-max values. The Mann-Whitney U test was used for statistical analyses. Abbreviations: VAS: visual analog scale; ODI: Oswestry disability index, Min: minimum, Max: maximum

EPIDURAL LYSIS (n: 72)						
Variables	Compared Endpoints (%)	PATIENT WITH LUMBAR SURGERY HISTORY (n:30)		PATIENT WITHOUT LUMBAR SURGERY HISTORY(n:42)		
		Median	Min-Max	Median	Min-Max	P-value
VAS	Pre-procedure-Post-procedure 1^st^ month	44.44	0-55.56	50	0-66.67	<0.001
	Pre-procedure-Post-procedure 6^th^ month	62.5	0-66.67	62.5	0-71.43	0.163
ODI	Pre-procedure-Post-procedure 1^st^ month	54.25	2.63-58.51	53.2	2.63-56.58	0.078
	Pre-procedure-Post-procedure 6^th^ month	61.8	2.63-65.96	62.9	2.63-64.52	0.473

The VAS and ODI scores decreased significantly in both patients with and without a history of lumbar surgery one month after the procedure compared to before the procedure and six months after the procedure compared to one month after the procedure and before the procedure (p<0.001). The paired temporal comparisons of the data of the patients who underwent epidural lysis procedures before the procedure, one month after the procedure, and six months after the procedure revealed significant differences in the VAS and ODI scores (p<0.001). Pregabalin, duloxetine, and opioid doses used by the patients one month and six months after the procedure decreased significantly compared to those before the procedure (p<0.001, p<0.001; p=0.018, p<0.001; p<0.001, p<0.001), whereas there was no significant difference between the doses used one month and six months after the procedure (p=1.00, p=0.401, p=0.589) (Table 4).

**Table 4:** Statistical comparison of variables assessed at the study’s endpoints Comparison of statistical data over time. Friedman tests were used for statistical analyses. Significance values have been adjusted by the Bonferroni correction for multiple tests (Bonferroni p=0.05/3=0.016). Endpoint 1: Pre-procedure, Endpoint 2: Post-procedure first month, Endpoint 3: Post-procedure sixth month a: adjusted P-value

Variables	Endpoints	Median	Median 1^st ^quartile	Median 3^rd^ quartile	P-value	(I) Time	(J) Time	Test Statistic	Standart Test Statistic	P-value	Adjusted P-value
VAS	1	8	7	8	<0.001	1	2	1.08	6.12	<0.001	<0.001^ a^
	2	4	3	4		2	3	0.68	5.75	<0.001	<0.001^ a^
	3	3	3	3		3	1	1.77	11.87	<0.001	<0.001^ a^
ODI	1	76	62	94	<0.001	1	2	1.02	6.12	<0.001	<0.001^ a^
	2	35	31	41.75		2	3	0.95	5.75	<0.001	<0.001^ a^
	3	29	25	35		3	1	1.97	11.87	<0.001	<0.001^ a^
Pregabalin Dose	1	150	37.5	150	<0.001	1	2	0.91	5.45	<0.001	<0.001^ a^
	2	75	18.75	150		2	3	0.11	0.70	0.479	1.000^ a^
	3	75	75	75		3	1	1.02	6.16	<0.001	<0.001^ a^
Opioid Dose	1	0	0	187.5	<0.001	1	2	0.45	2.75	0.006	0.018^ a^
	2	0	0	50		2	3	0.25	1.50	0.134	0.401^ a^
	3	0	0	0		3	1	0.70	4.25	<0.001	<0.001^ a^
Duloxetine Dose	1	30	0	60	<0.001	1	2	0.63	3.79	<0.001	<0.001^ a^
	2	0	0	30		2	3	0.21	1.29	0.196	0.589^ a^
	3	0	0	30		3	1	0.84	5.08	<0.001	<0.001^ a^

## Discussion

This study was conducted to evaluate the effectiveness of the epidural lysis procedure in patients with chronic radicular low back pain and filling defects on epidurogram. The findings of the study revealed significant decreases in the post-procedure one-month and six-month VAS and ODI scores of the patients with epidural fibrosis who underwent epidural lysis compared to those before the procedure. Additionally, pregabalin, duloxetine, and opioid doses used by the patients one month and six months after the procedure decreased significantly compared to the before the procedure. The analysis of the patients' VAS scores revealed that the VAS scores decreased significantly more one month after the procedure compared to those before the procedure in patients without a history of lumbar surgery than in patients with a history of lumbar surgery.

The methods used in the treatment of chronic lumbar radicular pain include medical analgesic drugs, centrally acting drugs (e.g., duloxetine and pregabalin, physical therapy and rehabilitation, lumbar epidural and transforaminal epidural steroid injections, dorsal root ganglion (DRG) pulse radiofrequency, and epidural lysis) [7]. A number of studies demonstrated the efficacy of epidural neuroplasty in the treatment of patients with persistent, chronic low back pain [8-11]. Percutaneous epidural neuroplasty is commonly performed in patients with refractory chronic low back pain or in whom back surgery syndrome has failed [12].

Epidural fibrosis-induced scar tissue fixes the nerve root in one position, causing inflammation of the nerve root. Inflammation causes stretching and compression of the nerve root and increased pain during movement [13]. An experimental animal model demonstrated that the changes in the affected nerve root, which are partly due to high phospholipase A2 levels initiated by inflammation in the nerve root, can be suppressed by epidural steroids [14]. Steroids administered caudally or interlaminarly do not distribute to the ventral epidural space in patients in whom the distribution of the contrast medium cannot be demonstrated. Failure of the drug to reach the epidural space is considered the primary reason for the failure of the treatment [13]. The objective of lysis is to eliminate adhesions in the epidural space and ensure that the anti-inflammatory drugs are distributed to the targeted ventral regions. A randomized, double-blind, prospective study comparing the efficacies of percutaneous lysis and epidural steroid injection treatments in patients with chronic lumbar radicular pain found a significant reduction (over 50%) in pain scores in more than 50% of the patients in the lysis group during the first six months compared to only 33% of the patients in the epidural steroid group during the first month. No pain relief was observed in any patient in the following months in the epidural steroid group. In addition, there was a statistically significant decrease in ODI scores in the lysis group [15]. Similarly, in our study, ODI and VAS scores one month and six months after the procedure decreased significantly compared to before the procedure.

After the epidural region is damaged due to surgery, multiple inflammatory and profibrotic cytokines such as platelet-derived growth factor (PDGF), transforming growth factor-beta 1 (TGF-β1), and insulin-like growth factor 1 (IGF-1) are excessively released, inducing the differentiation and migration of myofibroblasts into fibrotic foci. Activated fibroblasts can synthesize large amounts of extracellular matrix (ECM), leading to the formation of excessive scar tissues [16-18]. In our study, patients’ VAS scores decreased significantly more one month after the procedure compared to before the procedure in patients without a history of lumbar surgery than in patients with a history of lumbar surgery. On the other hand, there was no significant difference between the patients with and without a history of lumbar surgery in the percentage difference between patients’ pre-procedure VAS scores and VAS scores six months after the procedure. This finding may be attributed to the fact that surgery-related epidural fibrosis was more frequent and distributed to a larger area in patients with a history of lumbar surgery compared to patients without a history of lumbar surgery. In parallel, the more significant decrease in the VAS scores of patients without a history of lumbar surgery one month after the procedure compared to before the procedure than in those of patients with a history of lumbar surgery may be attributed to the rapid distribution of the anti-inflammatory drug given after the neuroplasty procedure in the epidural region. In our study, a statistically more significant decrease was observed in the group without a history of surgery when the difference between the pre-procedure and post-procedure one-month VAS scores was compared, as a percentage in the group with and without surgery, while no statistically significant difference was found when the difference between the pre-procedure and post-procedure six-month VAS scores was compared as a percentage. This may be attributed to the fact that the epidural fibrosis that developed after surgery was more and in a larger area than that in the non-surgical group. Therefore, this difference between the two groups in the first month after the procedure may be due to the rapid spread of the anti-inflammatory drug given after the neuroplasty procedure in the region and its rapid effect as a result. The absence of this difference at month six may be due to the effective spread of anti-inflammatory drugs in both groups, reaching an effective level and effective neural flossing exercise. Additional studies may be needed to investigate whether there are other reasons for the disappearance of this difference in both groups at six months. In addition, in our study, in parallel with the significant decreases in post-procedure VAS and ODI scores compared to before the procedure, there were significant decreases in the post-procedure opioid and pregabalin and duloxetine doses used by the patients compared to those before the procedure.

As with all invasive procedures, neuroplasty may cause complications, including bending of the needle tip, rupture of the catheter, blood aspiration and bleeding in the epidural space, misplacement or blocking of the catheter, hypotension, migration of the catheter/penetration of the catheter into the dura, paresthesia, headaches, infections, epidural abscess, meningitis, and cauda equina syndrome [19,20]. A meta-analysis study of five randomized, controlled studies and two observational studies reported that the incidence of complications from percutaneous adhesiolysis was lower than other similar procedures and that these complications were generally minimal and self-limiting [21]. Although rare, neuroplasty may cause serious side effects and complications in some cases. A case report published in 2023 reported a patient presented with paraplegia five days after the neuroplasty procedure. The imaging findings indicated that the patient had extensive epidural hematoma between the T12 and S1 vertebrae [22]. In comparison, in our study, the procedure was terminated due to complications of intrathecal passage in three patients and catheter rupture in one patient. No neurological pathology was observed in these patients during follow-up. In order to avoid complications, epidural neuroplasty should be considered in patients in whom conservative treatment methods failed and on the basis of a multidisciplinary evaluation. Considering the severity of the associated complications, the procedure should be performed by experienced specialist doctors and in well-equipped centers.

The primary limitation of our study was its retrospective design. Secondly, there was no long-term follow-up. Prospective large-scale randomized studies are needed to establish the efficacy of the caudal neuroplasty-adhesiolysis procedure.

## Conclusions

This study’s findings demonstrated the short-term efficacy of caudal lysis in patients with chronic low back pain resistant to conservative treatment methods. Caudal lysis featuring targeted steroids, analgesics, and hyaluronidase significantly alleviated the pain of patients with lumbar epidural fibrosis. Fluoroscopy-guided percutaneous epidural neuroplasty reduced pain and improved physical functions and quality of life. We concluded that percutaneous epidural neuroplasty is a safe and effective treatment method for patients with lumbar epidural fibrosis. However, the procedure should be performed by doctors trained and specialized in neuroplasty. In addition, we determined that epidural lysis provided better outcomes in patients without a history of lumbar surgery in the early period, possibly due to the extensive and large area fibrosis that developed after surgery. Considering that it is a minimally invasive method performed under local anesthesia with few complications, epidural neuroplasty can be repeated in cases where partial pain palliation is achieved.
